# Association between clinical outcomes and postoperative first metatarsal rotational alignment assessed by weight-bearing CT scan in hallux valgus

**DOI:** 10.3389/fsurg.2025.1682172

**Published:** 2025-11-14

**Authors:** Suwimol Prusmetikul, Jakrapong Orapin, Satetha Vasaruchapong, Tulyapruek Tawonsawatruk, Suphaneewan Jaovisidha, Rawee Manatrakul, Phatthawit Tangkittithaworn, Sukij Laohajaroensombat

**Affiliations:** 1 Department of Orthopedics, Faculty of Medicine, Ramathibodi Hospital, Mahidol University, Ratchathewi, Thailand; 2 Department of Orthopedics, Chakri Naruebodin Medical Institute, Bang Phli District, Samut Prakan, Thailand; 3 Department of Diagnostic and Therapeutic Radiology, Ramathibodi Hospital, Mahidol University, Bangkok, Thailand

**Keywords:** hallux valgus, first metatarsal pronation, weight-bearing CT scan, clinical outcome, postoperative

## Abstract

**Background:**

The significance of rotational deformity in the operative treatment of hallux valgus is growing. However, its impact on clinical outcomes remains inadequately explored. This study aims to investigate associations between residual rotational deformity and clinical outcomes following hallux valgus corrections.

**Methods:**

This retrospective study analysed 47 postoperative feet, using WBCT to measure first metatarsal rotation via the *α* angle. The AOFAS Hallux MTP-IP, VAS-FA, and FAOS scores were assessed using this parameter.

**Results:**

Patients with residual first metatarsal pronation demonstrated significantly poorer functions (84.14 ± 18.50; *P*-value = 0.04), other complaint subscales (78.78 ± 19.17; *P*-value = 0.03), and overall scores of the VAS-FA (82.93 ± 17.99; *P*-value = 0.04). A lower alignment subscale was observed in the AOFAS Hallux MTP-IP score (12.26 ± 3.49; *P*-value = 0.04), while other scales showed no significant differences between groups.

**Conclusion:**

Residual first metatarsal pronation is associated with poorer clinical outcomes as shown by the overall score, function, and other complaint subscales of the VAS-FA, as well as the alignment subscale of the AOFAS Hallux MTP-IP. These findings underscore the importance of correcting rotational deformity for optimal results. Nonetheless, given the retrospective design of this study with only postoperative assessments, causal inferences regarding the role of residual pronation cannot be established and should be interpreted cautiously.

## Introduction

The hallux valgus is characterised by medial deviation of the first metatarsal and lateral deviation of the proximal phalanx, significantly impacting the wellbeing of patients due to pain, functional limitations, discomfort wearing footwear, and altered gait patterns (1–3). Surgical intervention aims to correct these deformities and alleviate the symptoms, with expected improvements in pain, foot function, and overall quality of life. Radiographic parameters, including hallux valgus angle (HVA) and intermetatarsal angle (IMA), are pivotal in selecting optimal operative procedures and evaluating postoperative alignment (4).

While weight-bearing radiographs traditionally serve as the essential tool for preoperative assessment, in particular for the angular deformity of hallux valgus, in recent times, there has been an increase in emphasis on rotational deformity due to its association with postoperative recurrence (5–7). Common radiographic parameters such as the lateral edge shape of the first metatarsal head and tibial sesamoid position have been proposed to assess the severity of pronation but present challenges in measurement reliability issues, particularly in postoperative radiographs (6, 8–10). Weight-bearing computerised tomography (WBCT) scanning has emerged as a more accurate tool for assessing rotational deformity in hallux valgus (10). Conti et al. (7) demonstrated that improved correction of first metatarsal pronation following the modified Lapidus procedure was associated with better patient-reported outcomes and lower recurrence rates, emphasising the clinical relevance of rotational alignment. However, their study focused on changes in pronation and evaluated a single patient-reported outcome measure. In addition, the relationship between residual rotational deformity and specific clinical outcomes remains underexplored.

Our study evaluates postoperative residual first metatarsal pronation as a standalone parameter, addressing scenarios where preoperative WBCT may be unavailable. We also incorporate three validated clinical outcome scales to comprehensively capture pain, function, and quality of life, facilitating a more robust and multidimensional evaluation. Therefore, this study advances existing knowledge by examining how residual rotational deformities correlate with diverse patient-centred outcomes in the postoperative period. In addition, the association between traditional angular parameters on plain radiographs (HVA and IMA) and clinical outcomes was also evaluated. We hypothesised that residual first metatarsal pronation and angular deformities adversely affect these clinical outcomes. By addressing a gap in the literature, this study seeks to enhance the understanding of how postoperative anatomical alignment relates to patient-centred recovery measures.

## Materials and methods

This retrospective study received ethical approval from the Committee on Human Rights Related to Research Involving Human Subjects, Faculty of Medicine, Ramathibodi Hospital, Mahidol University (MURA2020/268), prior to obtaining data. Patients who had previously undergone hallux valgus correction without other foot operations were recruited from June to August 2020. Consecutive cases were operated on from October 2011 to December 2019 by a single foot and ankle orthopaedic surgeon (SL). Surgical procedures were chosen based on individual deformity components, such as scarf osteotomy for severe IMA and HVA, or the Lapidus procedure for degenerative changes or hypermobility of the first tarsometatarsal joint. Patients had to have undergone surgery at least 6 months before recruitment to ensure capability for full weight-bearing radiographs.

Weight-bearing plain films of the operated foot were obtained on the same date as the assessment of the clinical outcomes, and a weight-bearing CT scan was scheduled within 1 week (pedCAT, CurveBeam LLC, Warrington, PA; medium view, 0.3-mm slice thickness, 0.3-mm slice interval, 120 kVp, 22.62 mAs). For WBCT acquisition, patients are typically required to perform a one-leg stand on the scanned side, which allows focused imaging of the affected foot under functional loading conditions. This method ensures the acquisition of accurate, weight-bearing three-dimensional images that are vital for assessing postoperative foot alignment. Four readers—two orthopaedic surgeons (JO, SV) and two musculoskeletal radiologists (SJ, RM)—individually assessed all radiographs. Prior to the radiologic measurement, the readers were provided training for the method of assessment and the use our institutional picture archiving and communication system (Synapse version 5.0; FUJIFILM Medical System, USA) , and consensus was reached among all readers. Each assessor evaluated the radiographs separately and was blinded to patient identification.

### Assessment of radiographic parameters and defining the normal range

#### Weight-bearing plain film

**Figure 1:**
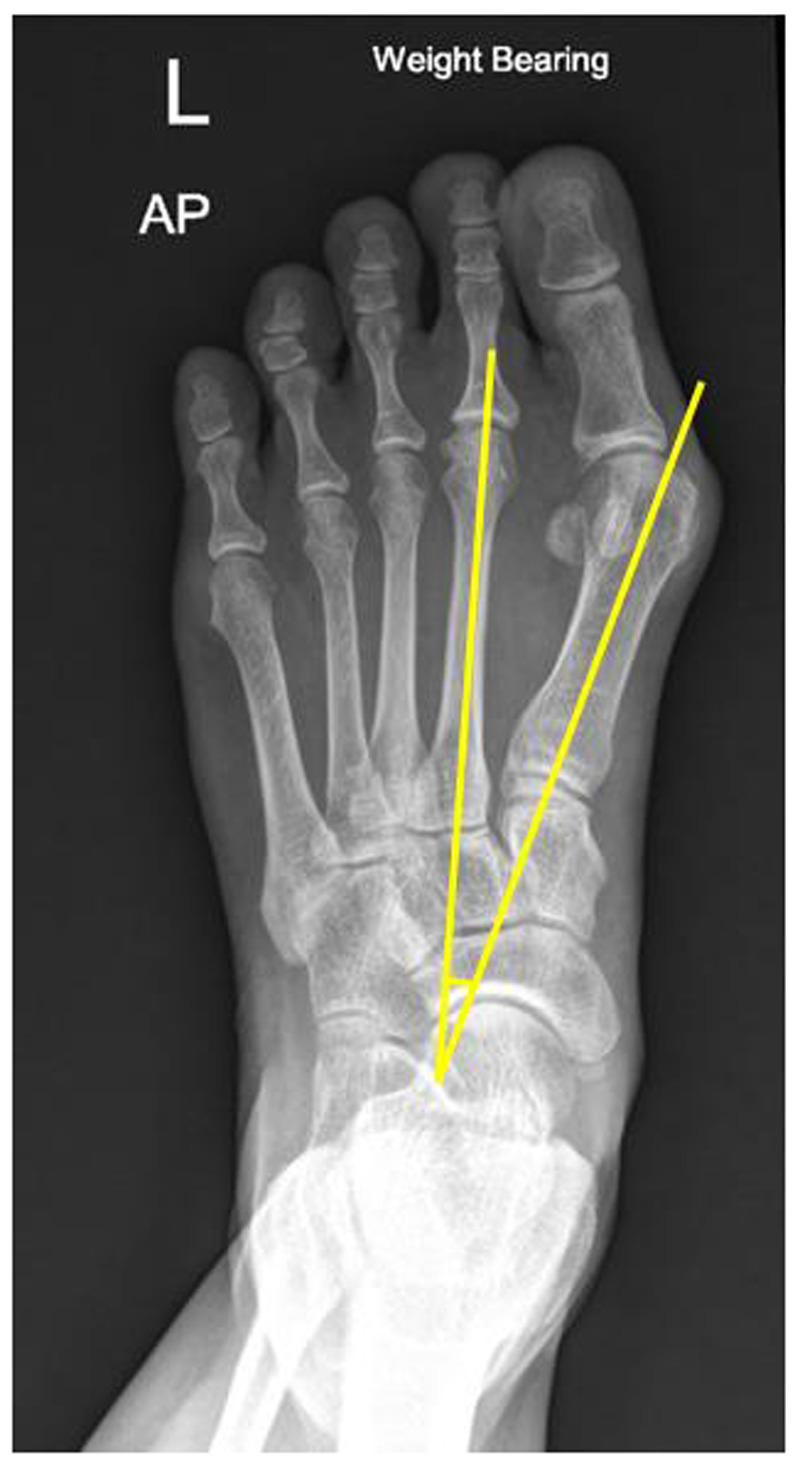
Illustration of IMA measurement in the weight-bearing plain film; the centres of the proximal and distal metaphyseal–diaphyseal areas of the 1st and 2nd metatarsal bones were marked as the axis of each bone. The intersection of the 1st and 2nd metatarsal axes was defined as the 1,2 intermetatarsal angle.

**Figure 2:**
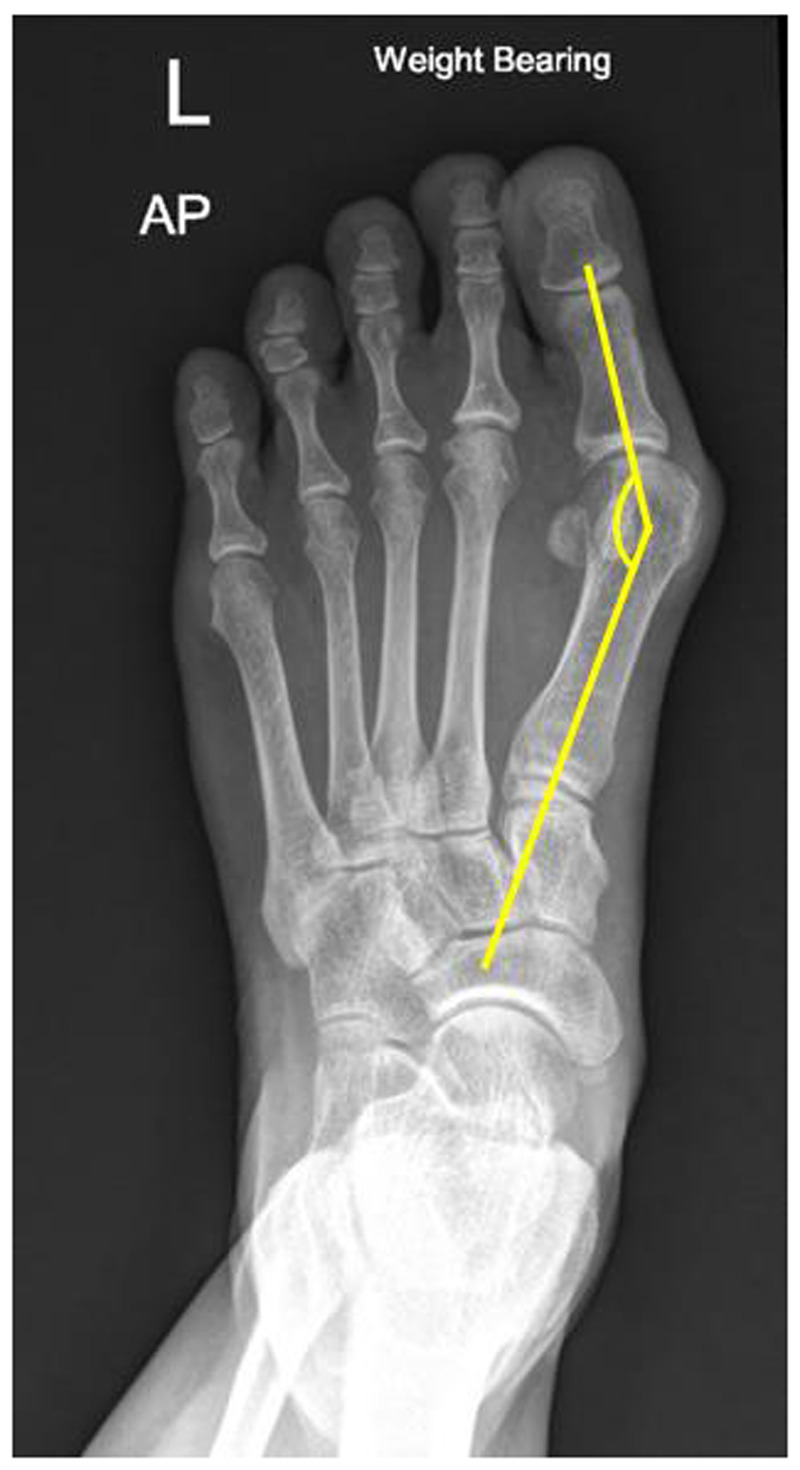
Illustration of HVA measurement in the weight-bearing plain film; the centres of the proximal and distal metaphyseal–diaphyseal areas of the proximal phalanx of the great toe were marked as the axis of the bone. The intersection of the 1st metatarsal and great toe proximal phalanx axes was defined as the hallux valgus angle.

A dorsoplantar view of the weight-bearing plain film was used to evaluate angular deformity. The measurement of parameters was performed based on the following standard methods:

1. 1,2 Intermetatarsal angle (IMA): The centres of the proximal and distal metaphyseal–diaphyseal areas of 1st and 2nd metatarsal bones were marked as the axis of each bone. The intersection of the 1st and 2nd metatarsal axes was defined as the IMA. An IMA of less than 9 degrees was considered normal (4) (Figure 1).
2. Hallux valgus angle (HVA): The centres of the proximal and distal metaphyseal–diaphyseal areas of the proximal phalanx of the hallux were marked as the axis of the bone. The intersection of the 1st metatarsal and hallux proximal phalanx axes was defined as the HVA. An HVA of less than 15 degrees was considered normal (4) (Figure 2).

#### Weight-bearing CT scan

**Figure 3:**
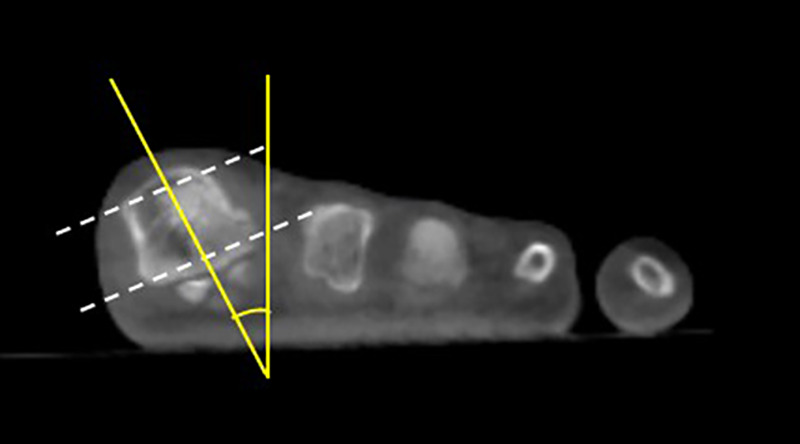
Illustration of *α* angle measurement in the weight-bearing CT scan; the dashed lines represent the referenced lines connecting the lateral and medial sulcus at the superior and inferior parts of the first metatarsal bone. The solid lines represent the *α* angle measurement as described.

Pronation of the first metatarsal bone was assessed by measuring the *α* angle, using the coronal plane of postoperative WBCT scans. The measurement was initiated by drawing two imaginary lines—inferior and superior lines (dashed lines in Figure 3). The inferior line was defined connecting the lateral edge of the lateral sulcus and the medial edge of the medial sulcus. Then, the superior line was drawn between the medial and lateral corners of the first metatarsal head. A straight line, which is used for measurement, was drawn connecting the centre of both inferior and superior lines; then, the angle was measured between this line and another vertical line perpendicular to the horizontal ground axis (solid lines in Figure 3).

The normal range of the *α* angle was defined as being between −4 and 18 degrees [representing two standard deviations (SDs) from the mean], in accordance with a study by Najefi et al. (11).

#### Assessment of clinical outcomes

Clinical outcomes were assessed separately for each foot in patients with a history of bilateral surgery. This approach was chosen because each foot may have undergone different surgical procedures and could demonstrate distinct postoperative hallux valgus parameters. Evaluating each side individually allows for a more accurate reflection of the clinical outcomes related to the angular variations of each foot. The details of each outcome assessment were evaluated based on the following:

1. American Orthopedic Foot and Ankle Society Hallux Metatarsophalangeal-Interphalangeal Scale (AOFAS Hallux MTP-IP)
  The AOFAS Hallux MTP-IP contains eight questions subdivided into three subscales of pain, function, and alignment. This scale is designed for subjective and objective assessments. A score of 100 points could be presented in patients with no pain, normal function, and good hallux alignment. A score of 0 points indicates severe pain, severe functional limitation, and poor alignment of the hallux (12).
2. Visual Analogue Scale Foot and Ankle (VAS-FA) (Thai version)
  The VAS-FA contains 20 items categorised into three subscales of pain, function, and other complaints, such as effects on daily activities. Each question ranges from 0 to 100 points, and patients can score subjectively. The score is categorised into a group of subscales. A score of 100 points represents no pain, good function, and none of other complaints. A score of 0 points defines severe pain, poor function, and other complaints (13).
3. Foot and Ankle Outcome Score (FAOS)
  The FAOS contains 42 questions subdivided into five subscales of symptoms, pain, activities of daily living, sports and recreational capacity, and quality of life. Each item is rated as none, mild, moderate, severe, or extreme. The score is reported corresponding to the category of questions. The best result of each subscale is 100 points, and the worst result is 0 (14).

All assessment tools demonstrated a good level of validity, reliability, or responsiveness (13–17). These assessments were used in multiple studies on hallux valgus. All clinical outcomes were evaluated by a single foot and ankle orthopaedic surgeon (SP).

#### Statistical analysis

An intraclass correlation coefficient (ICC) was calculated with absolute agreement using a two-way random effects model to investigate the intraobserver reliability (single measurement) and interobserver reliability (average measurement) of each parameter. Reliability was classified as follows: poor, ICC ≤ 0.20; fair, ICC = 0.21 to 0.40; moderate, ICC = 0.41 to 0.60; good, ICC = 0.61 to 0.80; and very good, ICC = 0.81 to 1.00. In addition to the ICC, Bland–Altman analysis was performed to evaluate the agreement and detect any systematic bias between repeated measurements. The mean difference and 95% limits of agreement were calculated and plotted.

The average measurement of each parameter among the four readers was determined in terms of mean and standard deviation (SD) values. The clinical outcomes were categorised into two groups based on the normal or abnormal range of each parameter as defined previously (normal IMA < 9 degrees, normal HVA < 15 degrees, and normal *α* angle is between −4 and 18 degrees) (4, 11).

The clinical outcomes (continuous variables) were compared between the normal and abnormal groups using Student's *t*-test. The assumption of normality for these continuous variables was evaluated using the Kolmogorov–Smirnov test. The significance of data was determined in terms of a *P*-value < .05 within a 95% confidence interval. Statistical analysis was performed using the SPSS statistical package (version 20.0.0; SPSS, Cary, NC).

## Results

### Demographic data

Thirty-three patients were recruited; 14 participants (42.42%) were operated upon and assessed bilaterally, resulting in a total of 47 feet (24 right; 23 left) evaluated via weight-bearing plain film and CT scan. All participants were female with a mean age of 49.3 years (SD, 17.13; range, 20–76 years) and a mean body mass index of 21.54 kg/m^2^ (SD, 2.51; range, 15.10–27.59 kg/m^2^). The patient's history of operative procedures included scarf in 33 feet (70.21%), Lapidus in 7 feet (14.89%), chevron in 6 feet (12.76%), first MTP joint arthrodesis in 1 ft (2.13%), and akin in 27 feet (57.45%). The mean follow-up period or interval between the operative date and clinical outcome evaluation was 49.81 months (SD, 32.72; range, 7–112 months). Both weight-bearing plain imaging and CT scans were performed within 1 week of clinical outcome evaluation.

### Reliability of measurement

**Figure 4:**
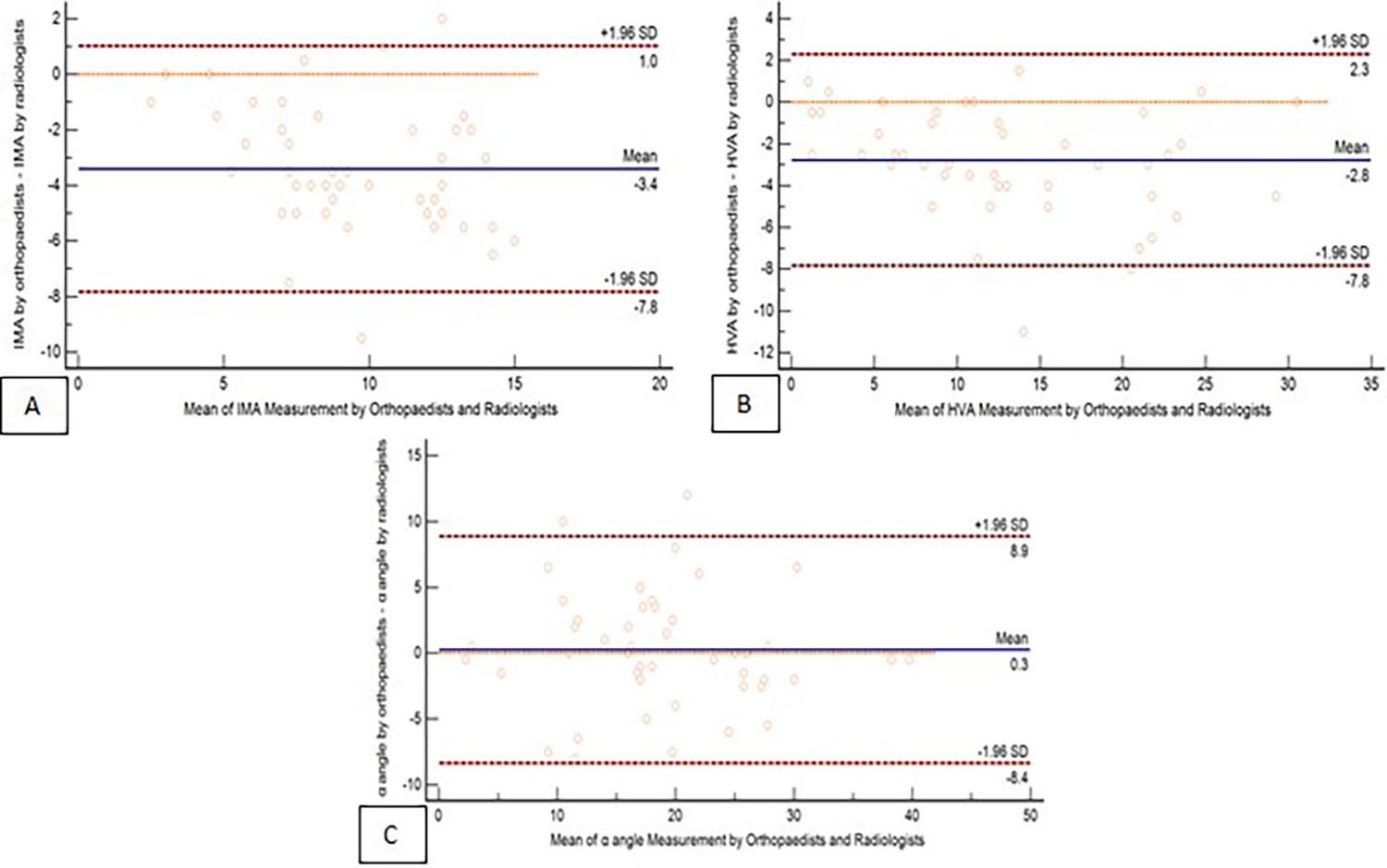
Interobserver reliability of parameter measurements: Bland–Altman plots comparing the parameter measurements obtained by orthopaedists and radiologists. The panels illustrate the agreement for three parameters: (**A**) IMA, (**B**) HVA, (**C**) *α* angle.

For interobserver reliability, the ICC of the IMA, HVA, and *α* angle were 0.83, 0.97, and 0.93, respectively. For intraobserver reliability, the ICC of the IMA, HVA, and *α* angle were 0.94, 0.98, and 0.83, respectively. All results corresponded with very high reliability (Table 1). The standard error measurement (SEM) range was between 0.84 and 3.29, and the minimal detectable change (MDC) was between 2.33 and 9.12. The Bland–Altman plots illustrate the interobserver agreement for all three parameters by plotting the difference between the measurements of the orthopaedists and radiologists against the mean of their measurements (Figure 4).

**Table 1:** The inter- and intraobserver reliability of parameters.

Parameters	Interobserver reliability				Intraobserver reliability			
	ICC	95% CI	SEM	MDC	ICC	95% CI	SEM	MDC
IMA	0.83	0.59–0.92	1.63	4.52	0.94	0.90–0.97	0.84	2.33
HVA	0.97	0.93–0.98	1.38	3.83	0.98	0.96–0.99	1.09	3.02
*α* Angle	0.93	0.90–0.96	2.34	6.49	0.83	0.66–0.91	3.29	9.12

ICC, intraclass correlation; CI, confidence interval; SEM, standard error measurement; MDC, minimal detectable change.

### The relation of parameters and clinical outcomes

#### *α* Angle

The mean *α* angle for all recruited feet was 18.97 degrees (SD, 8.05; range, 2–39 degrees). A normal *α* angle (≤18 degrees) was found in 24 feet, while an abnormal *α* angle was noted in 23 feet.

The clinical outcomes were compared between groups categorised by normal and abnormal *α* angles of the first metatarsal bone (Table 2). The alignment subscale of the AOFAS Hallux MTP-IP in the normal *α* angle group (mean, 14.13; SD, 2.36) was significantly better than that in the abnormal *α* angle group (mean, 12.26; SD, 3.49) (*P*-value = .04). However, the overall score and other subscales of the AOFAS Hallux MTP-IP were not significantly different between groups.

**Table 2:** Comparison of clinical outcomes between the groups of feet with normal and abnormal *α* angle.

Covariates	Normal *α* angle (*N* = 24)	Abnormal *α* angle (*N* = 23)	*P*-value	95% CI
**AOFAS Hallux MTP-IP, mean (SD)**				
Overall [100]	92.88 (5.39)	89.09 (7.65)	0.06	−7.66 to 0.09
Pain [40]	36.67 (4.82)	35.22 (5.93)	0.36	−4.62 to 1.72
Function [45]	42.08 (4.15)	41.61 (3.37)	0.67	−2.70 to 1.75
Alignment [15]	14.13 (2.36)	12.26 (3.49)	0.04*	−3.61 to −0.12
**VAS-FA, mean (SD)**				
Overall [100]	91.25 (4.61)	82.93 (17.99)	0.04*	−16.29 to −0.36
Pain [100]	92.20 (7.81)	85.87 (21.26)	0.19	−15.98 to 3.32
Function [100]	92.77 (5.77)	84.14 (18.50)	0.04*	−16.92 to −0.34
Other [100]	88.79 (8.47)	78.78 (19.17)	0.03*	−18.91 to −1.12
**FAOS, mean (SD)**				
Overall [100]	86.47 (6.35)	82.41 (17.03)	0.29	−11.80 to 3.68
Symptoms [100]	84.08 (12.01)	82.92 (17.63)	0.79	−9.99 to 7.67
Pain [100]	91.97 (6.03)	86.35 (14.33)	0.09	−12.23 to 0.99
Activities of daily living [100]	95.52 (6.80)	90.98 (17.34)	0.25	−12.47 to 3.40
Sports and recreational capacity [100]	80.83 (14.57)	76.25 (25.55)	0.46	−16.97 to 7.80
Quality of life [100]	79.95 (15.20)	75.54 (24.20)	0.46	−16.41 to 7.60

[ ] = full score of the particular domain.

*Data that are statistically significant difference.

In the VAS-FA, the normal *α* angle group (mean, 91.25; SD, 4.61) showed significantly better overall scores than the abnormal *α* angle group (mean, 82.93; SD, 17.99) (*P*-value = .04). The function subscale of the normal *α* angle group (mean, 92.77; SD, 5.77) also had a significantly better score than that of the abnormal *α* angle group (mean, 84.14; SD, 18.50) (*P*-value = .04). In addition, the normal *α* angle group demonstrated a significantly better score in the other complaint subscale compared to the abnormal *α* angle group (mean, 88.79; SD, 8.47; and mean, 78.78; SD, 19.17, respectively) (*P*-value = .03). However, the pain subscale was not significantly different between groups.

Finally, the overall score and subscales of the FAOS did not show a statistically significant difference between groups.

#### IMA

The mean IMA of all recruited feet was 9.53 degrees (SD, 3.15; range 2–15 degrees). A normal IMA (<9 degrees) was found in 22 feet (46.81%), and an abnormal IMA was found in 25 feet (53.19%).

The AOFAS Hallux MTP-IP, VAS-FA, and FAOS were compared between groups with normal and abnormal IMAs (Table 3). The total score and subscales of all clinical outcomes were not significantly different between groups.

**Table 3:** Comparison of clinical outcomes between the groups of feet with normal and abnormal IMA.

Covariates	Normal IMA (*N* = 22)	Abnormal IMA (*N* = 25)	*P*-value	95% CI
**AOFAS Hallux MTP-IP, mean (SD)**				
Overall [100]	90.77 (7.16)	91.24 (6.60)	0.82	−3.58 to 4.51
Pain [40]	35.91 (5.90)	36.00 (5.00)	0.95	−3.11 to 3.29
Function [45]	41.14 (4.06)	42.48 (3.42)	0.22	−0.85 to 3.54
Alignment [15]	13.73 (2.76)	12.76 (3.33)	0.29	−2.78 to 0.85
**VAS-FA, mean (SD)**				
Overall [100]	87.38 (10.99)	87.00 (15.65)	0.92	−8.43 to 7.67
Pain [100]	90.19 (17.21)	88.14 (15.21)	0.66	−11.58 to 7.47
Function [100]	89.84 (11.12)	87.41 (16.46)	0.56	−10.80 to 5.94
Other [100]	82.10 (11.65)	85.46 (18.18)	0.45	−5.54 to 12.24
**FAOS, mean (SD)**				
Overall [100]	84.48 (12.07)	84.48 (13.60)	0.99	−7.60 to 7.60
Symptoms [100]	87.18 (11.80)	80.28 (16.69)	0.11	−15.50 to 1.71
Pain [100]	89.97 (11.61)	88.55 (10.94)	0.67	−8.05 to 5.21
Activities of daily living [100]	90.72 (13.69)	95.58 (12.43)	0.21	−2.82 to 12.54
Sports and recreational capacity [100]	75.85 (20.91)	81.00 (20.41)	0.40	−7.01 to 17.30
Quality of life [100]	78.69 (19.45)	77.00 (20.86)	0.78	−13.60 to 10.21

[ ] = full score of the particular domain.

#### HVA

The overall mean HVA was 13.26 degrees (SD, 7.52; range 1–30 degrees). When dividing into groups, the normal HVA group (<15 degrees) had 30 feet (63.83%) and the and abnormal HVA group had 17 feet (36.17%).

The alignment subscale of the AOFAS Hallux MTP-IP in the normal HVA group (mean, 14.53; SD, 1.78) was significantly better than that in the abnormal HVA group (mean, 10.88; SD, 3.55) (*P*-value = .0007). However, the overall score and other subscales of the AOFAS Hallux MTP-IP, as well as the overall score and subscales of the VAS-FA and FAOS, did not show a statistically significant difference between groups (Table 4).

**Table 4:** Comparison of clinical outcomes between the groups of feet with normal and abnormal HVA.

Covariates	Normal HVA (*N* = 30)	Abnormal HVA (*N* = 17)	*P*-value	95% CI
**AOFAS Hallux MTP-IP, mean (SD)**				
Overall [100]	92.03 (7.09)	89.24 (6.03)	0.18	−6.91 to 1.32
Pain [40]	35.67 (5.68)	36.47 (4.93)	0.63	−2.51 to 4.12
Function [45]	41.83 (3.82)	41.88 (3.74)	0.97	−2.27 to 2.37
Alignment [15]	14.53 (1.78)	10.88 (3.55)	0.0007*	−5.56 to −1.74
**VAS-FA, mean (SD)**				
Overall [100]	89.20 (10.95)	83.62 (16.96)	0.24	−15.01 to 3.87
Pain [100]	90.86 (15.30)	85.99 (17.28)	0.32	−14.66 to 4.94
Function [100]	90.57 (11.09)	84.98 (18.14)	0.26	−15.61 to 4.43
Other [100]	86.15 (12.72)	79.89 (19.03)	0.18	−15.60 to 3.07
**FAOS, mean (SD)**				
Overall [100]	85.37 (10.94)	82.91 (15.75)	0.53	−10.32 to 5.40
Symptoms [100]	85.48 (12.65)	80.04 (18.04)	0.23	−14.48 to 3.61
Pain [100]	89.22 (10.58)	89.21 (12.45)	0.99	−6.90 to 6.89
Activities of daily living [100]	93.04 (12.46)	93.77 (14.62)	0.86	−7.38 to 8.85
Sports and recreational capacity [100]	78.29 (17.35)	79.12 (25.93)	0.90	−11.90 to 13.55
Quality of life [100]	80.83 (18.92)	72.43 (21.32)	0.17	−20.52 to 3.70

[ ] = full score of the particular domain.

*Data that are statistically significant difference.

Following the presentation of the clinical outcomes for the entire cohort, we further analysed these results by subgroup according to whether patients underwent unilateral or bilateral hallux valgus surgery. This subgroup analysis aimed to explore potential differences in postoperative function and patient-reported outcomes between these patients.

### Outcomes of patients with unilateral operation (19 feet)

#### *α* Angle

The mean *α* angle for all feet of patients who had unilateral operation was 19.17 degrees (SD, 6.79; range, 9–30 degrees). A normal *α* angle (≤18 degrees) was found in 8 feet (42.11%), while an abnormal *α* angle was defined in 11 feet (57.89%).

The clinical outcomes were compared between groups categorised by normal and abnormal *α* angles of the first metatarsal bone (Table 5). The pain subscale of the AOFAS Hallux MTP-IP in the normal *α* angle group (mean, 38.75; SD, 3.54) was significantly better than that in the abnormal *α* angle group (mean, 32.73; SD, 6.47) (*P*-value = .03). The overall AOFAS Hallux MTP-IP was also significantly better in the normal *α* angle group (mean, 95.38; SD, 5.71) compared with the abnormal *α* angle group (mean, 85.55; SD, 8.27) (*P*-value = .01). However, the function and alignment subscales were not significantly different between groups.

**Table 5:** Comparison of clinical outcomes between the normal and abnormal *α* angle following unilateral operation (*N* = 19).

Covariates	Normal *α* angle (*N* = 8)	Abnormal *α* angle (*N* = 11)	*P*-value	95% CI
**AOFAS Hallux MTP-IP, mean (SD)**				
Overall [100]	95.38 (5.71)	85.55 (8.27)	0.01*	−17.01 to −2.65
Pain [40]	38.75 (3.54)	32.73 (6.47)	0.03*	−11.37 to −0.68
Function [45]	42.50 (3.78)	39.73 (3.29)	0.11	−6.20 to 0.66
Alignment [15]	14.13 (2.47)	13.09 (3.27)	0.46	−3.94 to 1.88
**VAS-FA, mean (SD)**				
Overall [100]	92.19 (5.76)	73.89 (21.58)	0.02*	−33.15 to −3.45
Pain [100]	91.47 (11.44)	76.39 (27.33)	0.12	−34.77 to 4.61
Function [100]	91.85 (8.30)	73.87 (21.60)	0.02*	−33.30 to −2.65
Other [100]	93.25 (5.39)	71.38 (22.58)	0.009*	−37.27 to −6.46
**FAOS, mean (SD)**				
Overall [100]	85.90 (7.25)	72.99 (19.76)	0.07	−26.91 to 1.11
Symptoms [100]	79.91 (14.40)	77.27 (22.43)	0.77	−21.79 to 16.51
Pain [100]	91.67 (5.35)	77.53 (15.29)	0.01*	−24.91 to −3.38
Activities of daily living [100]	95.74 (9.11)	83.16 (22.09)	0.11	−28.45 to 3.29
Sports and recreational capacity [100]	79.38 (19.35)	59.43 (25.66)	0.08	−42.76 to 2.87
Quality of life [100]	82.81 (12.83)	67.61 (28.20)	0.14	−35.74 to 5.34

[ ] = full score of the particular domain.

*Data that are statistically significant difference.

In the VAS-FA, the normal *α* angle group (mean, 92.19; SD, 5.76) showed a significantly better overall score than the abnormal *α* angle group (mean, 73.89; SD, 21.58) (*P*-value = .02). The function subscale of the normal *α* angle group (mean, 91.85; SD, 8.30) had a significantly better score than that of the abnormal *α* angle group (mean, 73.87; SD, 21.60) (*P*-value = .02). In addition, the normal *α* angle group (mean, 93.25; SD, 5.39) demonstrated a significantly better score in the other complaint subscale compared to the abnormal *α* angle group (mean, 71.38; SD, 22.58) (*P*-value = .009). However, the pain subscale was not significantly different between groups.

Only the pain subscale of the FAOS in the normal *α* angle group (mean, 91.67; SD, 5.35) was significantly better than that of the abnormal *α* angle group (mean, 77.53; SD, 15.29) (*P*-value = .01), while the overall score and other subscales did not show a statistically significant difference between groups.

#### IMA

The mean IMA was 10.07 degrees (SD, 3.12; range 4–15 degrees). A normal IMA (<9 degrees) was found in 7 feet (36.84%), and an abnormal IMA was found in 12 feet (63.16%).

The AOFAS Hallux MTP-IP, VAS-FA, and FAOS were compared between groups with normal and abnormal IMAs (Table 6). The total score and subscales of all clinical outcomes were not significantly different between groups.

**Table 6:** Comparison of clinical outcomes between the normal and abnormal IMA following unilateral operation (*N* = 19).

Covariates	Normal IMA (*N* = 7)	Abnormal IMA (*N* = 12)	*P*-value	95% CI
**AOFAS Hallux MTP-IP, mean (SD)**				
Overall [100]	86.86 (9.44)	91.33 (8.18)	0.29	−4.20 to 13.15
Pain [40]	32.86 (7.56)	36.67 (4.92)	0.20	−2.2 to 9.82
Function [45]	40.00 (2.89)	41.42 (4.10)	0.43	−2.31 to 5.15
Alignment [15]	14.00 (2.65)	13.25 (3.17)	0.61	−3.75 to 2.25
**VAS-FA, mean (SD)**				
Overall [100]	79.30 (16.76)	82.93 (20.66)	0.70	−15.81 to 23.07
Pain [100]	81.64 (29.08)	83.38 (19.89)	0.88	−21.90 to 25.36
Function [100]	79.29 (14.12)	82.70 (22.15)	0.72	−16.36 to 23.17
Other [100]	76.94 (16.23)	82.72 (22.94)	0.57	−15.12 to 26.67
**FAOS, mean (SD)**				
Overall [100]	76.86 (16.78)	79.35 (17.39)	0.76	−14.75 to 19.73
Symptoms [100]	86.73 (16.59)	73.51 (19.29)	0.15	−31.67 to 5.22
Pain [100]	81.35 (15.53)	84.72 (13.37)	0.62	−10.85 to 17.59
Activities of daily living [100]	80.85 (20.25)	92.89 (16.77)	0.18	−6.11 to 30.17
Sports and recreational capacity [100]	61.25 (26.78)	71.67 (23.87)	0.39	−14.60 to 35.44
Quality of life [100]	74.11 (22.94)	73.96 (25.26)	0.99	−24.70 to 24.40

[ ] = full score of the particular domain.

#### HVA

The mean HVA was 13.68 degrees (SD, 7.97; range 1–29 degrees). A normal HVA (<15 degrees) was found in 11 feet (57.89%), and an abnormal IMA was found in 8 feet (42.11%).

The overall score and all subscales of the AOFAS Hallux MTP-IP, VAS-FA, and FAOS did not demonstrate a significant difference between groups with normal and abnormal HVAs (Table 7).

**Table 7:** Comparison of clinical outcomes between the normal and abnormal HVA following unilateral operation (*N* = 19).

Covariates	Normal HVA (*N* = 11)	Abnormal HVA (*N* = 8)	*P*-value	95% CI
**AOFAS Hallux MTP-IP, mean (SD)**				
Overall [100]	88.73 (8.06)	89.25 (8.51)	0.89	−7.57 to 8.61
Pain [40]	33.64 (6.74)	37.50 (4.63)	0.18	−1.98 to 9.71
Function [45]	41.36 (3.23)	40.25 (4.37)	0.53	−4.78 to 2.55
Alignment [15]	13.73 (2.83)	11.50 (3.74)	0.16	−5.40 to 0.95
**VAS-FA, mean (SD)**				
Overall [100]	84.73 (16.73)	77.27 (21.98)	0.41	−26.15 to 11.23
Pain [100]	84.23 (23.52)	80.69 (23.45)	0.75	−26.57 to 19.49
Function [100]	84.34 (15.82)	77.45 (23.67)	0.46	−25.95 to 12.17
Other [100]	85.62 (18.19)	73.68 (22.51)	0.22	−31.63 to 7.75
**FAOS, mean (SD)**				
Overall [100]	81.19 (15.14)	74.64 (19.10)	0.42	−23.10 to 10.00
Symptoms [100]	85.07 (16.74)	69.19 (19.08)	0.07	−33.26 to 1.52
Pain [100]	82.83 (13.37)	84.38 (15.42)	0.82	−12.42 to 15.52
Activities of daily living [100]	88.61 (18.19)	88.24 (20.30)	0.97	−19.09 to 18.34
Sports and recreational capacity [100]	69.89 (19.97)	65.00 (31.51)	0.68	−29.75 to 19.98
Quality of life [100]	79.55 (23.06)	66.41 (24.08)	0.25	−36.16 to 9.89

[ ] = full score of the particular domain.

#### Outcomes of patients with bilateral operation (28 feet)

This group of patients underwent bilateral hallux valgus corrections. Four patients (8 feet) had simultaneous bilateral operations, while the remaining 10 patients (20 feet) underwent staged bilateral procedures on different dates. Clinical outcomes were assessed separately for each foot, as residual angular parameters could vary between sides.

#### *α* Angle

The mean *α* angle for all feet of patients who had bilateral operation was 18.57 degrees (SD, 8.91; range, 2–39 degrees). A normal *α* angle (≤18 degrees) was found in 17 feet (60.71%), while an abnormal *α* angle was defined in 11 feet (39.29%).

The clinical outcomes were compared between groups categorised by normal and abnormal *α* angles (Table 8). The alignment subscale of the AOFAS Hallux MTP-IP in the normal *α* angle group (mean, 14.18; SD, 2.32) was significantly better than that of the abnormal *α* angle group (mean, 11.18; SD, 3.66) (*P*-value = .01). However, the overall score and other subscales did not present a significant difference between groups.

**Table 8:** Comparison of clinical outcomes between the normal and abnormal *α* angle following bilateral operation (evaluated each side separately) (*N* = 28).

Covariates	Normal *α* angle (*N* = 17)	Abnormal *α* angle (*N* = 11)	*P*-value	95% CI
**AOFAS Hallux MTP-IP, mean (SD)**				
Overall [100]	91.53 (4.80)	92.55 (5.77)	0.62	−3.11 to 5.15
Pain [40]	35.29 (5.15)	38.18 (4.05)	0.13	−0.89 to 6.67
Function [45]	42.06 (4.35)	43.18 (2.52)	0.45	−1.86 to 4.11
Alignment [15]	14.18 (2.32)	11.18 (3.66)	0.01*	−5.31 to −0.68
**VAS-FA, mean (SD)**				
Overall [100]	90.51 (4.08)	91.68 (8.43)	0.68	−4.73 to 7.06
Pain [100]	91.96 (6.02)	95.68 (6.45)	0.13	−1.19 to 8.65
Function [100]	92.97 (4.27)	93.99 (7.85)	0.70	−4.51 to 6.56
Other [100]	86.62 (8.69)	85.36 (13.52)	0.77	−9.85 to 7.34
**FAOS, mean (SD)**				
Overall [100]	87.14 (6.10)	90.84 (7.76)	0.17	−1.70 to 9.10
Symptoms [100]	86.98 (10.71)	87.01 (9.88)	0.99	−8.23 to 8.31
Pain [100]	92.58 (6.58)	93.94 (7.12)	0.61	−4.04 to 6.76
Activities of daily living [100]	95.69 (5.60)	97.99 (6.66)	0.33	−2.49 to 7.10
Sports and recreational capacity [100]	81.76 (11.85)	92.27 (13.30)	0.04*	0.62 to 20.39
Quality of life [100]	78.68 (15.94)	82.95 (18.98)	0.53	−9.38 to 17.94

[ ] = full score of the particular domain.

*Data that are statistically significant difference.

The overall score and all subscales of the VAS-FA were not significantly different between groups.

In the FAOS, the sports and recreational capacity subscale of the abnormal *α* angle group (mean, 92.27; SD, 13.30) was significantly better than that of the normal *α* angle group (mean, 81.76; SD, 11.85) (*P*-value = .04), while the overall score and remaining subscales did not show a statistically significant difference between groups.

#### IMA

The mean IMA was 9.15 degrees (SD, 3.21; range 2–14 degrees). A normal IMA (<9 degrees) was found in 15 feet (53.57%), and an abnormal IMA was found in 13 feet (46.43%).

The AOFAS Hallux MTP-IP, VAS-FA, and FAOS were compared between groups with normal and abnormal IMAs (Table 9). The total score and subscales of all clinical outcomes were not significantly different between groups.

**Table 9:** Comparison of clinical outcomes between the normal and abnormal IMA following bilateral operation (evaluated each side separately) (*N* = 28).

Covariates	Normal IMA (*N* = 15)	Abnormal IMA (*N* = 13)	*P*-value	95% CI
**AOFAS hallux MTP-IP, mean (SD)**				
Overall [100]	92.60 (5.25)	91.15 (5.06)	0.47	−5.47 to 2.58
Pain [40]	37.33 (4.58)	35.38 (5.19)	0.30	−5.74 to 1.84
Function [45]	41.67 (4.50)	43.46 (2.40)	0.19	−0.98 to 4.57
Alignment [15]	13.60 (2.90)	12.31 (3.54)	0.30	−3.79 to 1.21
**VAS-FA, mean (SD)**				
Overall [100]	91.15 (3.62)	90.76 (8.17)	0.88	−5.59 to 4.81
Pain [100]	94.18 (5.32)	92.54 (7.49)	0.50	−6.64 to 3.35
Function [100]	94.77 (4.30)	91.76 (7.02)	0.18	−7.46 to 1.46
Other [100]	84.51 (8.43)	87.98 (12.81)	0.40	−4.84 to 11.79
**FAOS, mean (SD)**				
Overall [100]	88.04 (7.43)	89.22 (6.50)	0.66	−4.28 to 6.65
Symptoms [100]	87.38 (9.53)	86.54 (11.31)	0.83	−8.94 to 7.25
Pain [100]	93.99 (6.65)	92.09 (6.88)	0.46	−7.17 to 3.35
Activities of daily living [100]	95.32 (5.86)	98.06 (6.11)	0.24	−1.91 to 7.40
Sports and recreational capacity [100]	82.67 (13.87)	89.62 (11.98)	0.17	−3.20 to 17.10
Quality of life [100]	80.83 (18.06)	79.81 (16.37)	0.88	−14.50 to 12.45

[ ] = full score of the particular domain.

#### HVA

The mean HVA was 12.89 degrees (SD, 7.53; range 1–30 degrees). A normal HVA (<15 degrees) was found in 19 feet (67.86%), and an abnormal IMA was found in 9 feet (32.14%).

The alignment subscale of the AOFAS Hallux MTP-IP in the group with the normal HVA (mean, 14.26; SD, 2.21) had a significantly better score than that of the abnormal HVA group (mean, 10.33; SD, 3.50) (*P*-value = .001). The overall score and the remaining subscales of the AOFAS Hallux MTP-IP did not demonstrate a statistically significant difference between groups (Table 10). The VAS-FA and FAOS were also not significantly different between groups in the overall score and all subscales.

**Table 10:** Comparison of clinical outcomes between the normal and abnormal HVA following bilateral operation (evaluated each side separately) (*N* = 28).

Covariates	Normal HVA (*N* = 19)	Abnormal HVA (*N* = 9)	*P*-value	95% CI
**AOFAS hallux MTP-IP, mean (SD)**				
Overall [100]	93.21 (5.46)	89.22 (3.03)	0.05	−8.02 to 0.04
Pain [40]	36.84 (4.78)	35.56 (5.27)	0.52	−5.39 to 2.82
Function [45]	42.11 (4.19)	43.33 (2.50)	0.43	−1.89 to 4.35
Alignment [15]	14.26 (2.21)	10.33 (3.50)	0.001*	−6.15 to −1.71
**VAS-FA, mean (SD)**				
Overall [100]	91.78 (4.31)	89.27 (8.75)	0.43	−9.38 to 4.35
Pain [100]	94.70 (5.20)	90.72 (7.95)	0.12	−9.12 to 1.16
Function [100]	94.18 (4.65)	91.67 (7.80)	0.30	−7.33 to 2.32
Other [100]	86.46 (8.75)	85.41 (14.41)	0.81	−10.05 to 7.94
**FAOS, mean (SD)**				
Overall [100]	87.80 (6.97)	90.27 (6.88)	0.39	−3.30 to 8.24
Symptoms [100]	85.71 (10.10)	89.68 (10.48)	0.35	−4.53 to 12.47
Pain [100]	92.92 (6.44)	93.51 (7.60)	0.83	−5.08 to 6.27
Activities of daily living [100]	95.59 (6.87)	98.69 (2.98)	0.11	−0.74 to 6.93
Sports and recreational capacity [100]	83.16 (13.97)	91.67 (10.00)	0.11	−2.20 to 19.22
Quality of life [100]	81.58 (16.73)	77.78 (18.25)	0.59	−18.12 to 10.51

[ ] = full score of the particular domain.

*Data that are statistically significant difference.

## Discussion

This study investigated the relationship between postoperative clinical outcomes and radiographic parameters in the treatment of hallux valgus, focusing on the *α* angle representing first metatarsal pronation. The results demonstrated that abnormal first metatarsal pronation correlated with significantly lower scores in the alignment subscale of the AOFAS Hallux MTP-IP, as well as decreased function, other complaints, and an overall lower score of the VAS-FA. However, no significant differences were observed in the overall score or other subscales of the AOFAS Hallux MTP-IP (pain and function), pain subscale of the VAS-FA, and all scales of the FAOS. For the transverse plane parameters, only the alignment subscale of the AOFAS Hallux MTP-IP was significantly lower in patients with an abnormal HVA; the other subscales of the AOFAS Hallux MTP-IP, all scales of the VAS-FA, and all scales of the FAOS showed no significant differences between normal and abnormal HVAs. Finally, no statistically significant difference in any clinical outcomes was found between patients with normal and abnormal postoperative IMAs.

By analysing outcomes separately for patients who underwent unilateral and bilateral operations, we observed both similarities and differences compared to the overall analysis. In the unilateral operation group, the pain subscales of the AOFAS Hallux MTP-IP and FAOS showed better scores in the normal *α* angle group, although only the AOFAS Hallux MTP-IP demonstrated a superior overall score. For the VAS-FA, the function and other complaint subscales, along with the overall score, were better in the normal *α* angle group, consistent with the results from the entire cohort.

In the bilateral operation group, the alignment subscale of the AOFAS Hallux MTP-IP was significantly better in the postoperative normal *α* angle and HVA. Interestingly, feet with an abnormal *α* angle showed better scores in the sports and recreational capacity subscale of the FAOS. This nuanced analysis highlights the differential impact of residual angular deformities on clinical outcomes depending on whether a unilateral or bilateral operation was performed. However, it is important to acknowledge that the alignment subscale of the AOFAS Hallux MTP-IP, although widely used, relies on single-physician evaluation and carries inherent limitations, including potential variability in scoring due to subjective assessment by individual clinicians.

Radiographic parameters play a crucial role in hallux valgus clinical practice and management, guiding surgical goals and postoperative monitoring for deformity recurrence (4). However, achieving satisfactory clinical outcomes is paramount for successful treatment. In our study, abnormal postoperative first metatarsal pronation was associated with poorer outcomes, particularly lower overall VAS-FA, including function and other complaints. In contrast, the pain subscale showed no significant difference, suggesting that residual pronation may contribute to functional limitations or discomfort during activities without notable pain. Contrarily, the AOFAS Hallux MTP-IP and FAOS subscales related to pain or function did not significantly differ between normal and abnormal rotation. Only the alignment subscale of the AOFAS Hallux MTP-IP was lower in patients with abnormal pronation, similar to an abnormal HVA, suggesting that a potentially notable hallux malalignment could occur postoperatively if these parameters are not corrected. As such, it could possibly lead to a remaining visible deformity or difficulty in wearing footwear. Other subscales of the AOFAS Hallux MTP-IP and all scales of the VAS-FA and FAOS showed no significant differences between normal and abnormal HVAs. These opposing results suggest that a residual postoperative HVA may relate to some degree of hallux malalignment clinically without pain or functional limitations. However, abnormal first metatarsal pronation and HVA exhibited associations with poorer clinical outcomes, while an abnormal IMA showed no such association.

Literature comparing postoperative clinical outcomes based on achieving normal radiographic parameters remains limited in studies on hallux valgus. Most prior investigations have focused on overall clinical improvement after surgery, with few examining correlations between specific radiographic parameters and functional outcomes. Nishikawa et al. (18) demonstrated improvements in the Short-Form 12 Health Survey (SF-12) and the Lower Extremity Functional Scale (LEFS) after a Lapidus procedure, finding an inverse correlation between IMA reduction and the physical scale of SF-12, as well as LEFS. No correlation was found between the change in HVA and clinical outcomes. Motta et al. (19) reported significant improvements in radiographic parameters, AOFAS Hallux MTP-IP scores, and Manchester–Oxford Foot Questionnaire (MOXFQ) outcomes following operation, without significant correlations between HVA, IMA changes, and clinical outcomes. Matthews et al. (20) found only weak correlations between postoperative radiographic parameters, including HVA and IMA, and FAOS subscales. The strongest association were observed between the IMA and the sports and recreation subscale (*r* = −0.33; *P*-value = .005), and between metatarsal protrusion distance and the function/daily living subscale (*r* = 0.33; *P*-value = .005). These findings highlight a recurring discrepancy between radiographic correlation and clinical outcomes, emphasising the complex clinical pathology of hallux valgus, where rotational deformities, soft tissue involvement, and dynamic foot function may influence recovery beyond static angular measures alone. The inconsistency between radiographic parameters and patient-reported outcomes suggests that anatomical correction does not uniformly translate to functional improvement. Our study reinforces this pattern. Postoperative normal and abnormal ranges of HVA and IMA showed no significant differences in the AOFAS Hallux MTP-IP, VAS-FA, and FAOS, except reduced alignment subscale scores in patients with abnormal HVAs, suggesting limited predictive value of these angular measures alone. In summary, while radiographic correction remains a pillar of hallux valgus treatment evaluation, our findings and the existing literature indicate that its direct translation into clinical benefit is complex and multifactorial. Future research should incorporate dynamic assessments, consider rotational deformities comprehensively, and comparatively analyse surgical techniques to elucidate factors predictive of successful outcomes.

The pathological and anatomical mechanisms underlying the contribution of first metatarsal rotational deformity to impaired function and pain in hallux valgus are pivotal to understanding the deformity. The hallux valgus involves transverse plane subluxation of the first metatarsophalangeal joint, commonly linked to abnormal pronation of the first metatarsal. This rotational deformity leads to altered biomechanical forces during gait, producing a valgus torque on the hallux and medial displacement of the first metatarsal, which cause joint instability and abnormal loading patterns. The medial collateral ligament and sesamoid ligaments play critical roles in resisting these abnormal motions. The failure of these static stabilizers contributes to progressive deformity. In addition, lateral displacement of the flexor hallucis longus tendon creates a force couple that exacerbates valgus alignment of the distal phalanx and medial drift of the first metatarsal (21, 22). The resulting joint malalignment and altered tendon vector forces could lead to impaired function, increased pain, and diminished patient outcomes. These biomechanical and anatomical insights underscore the clinical importance of correcting the first metatarsal rotational deformity to restore joint stability and improve function after hallux valgus surgery.

First metatarsal pronation is a prevalent component in hallux valgus deformities and plays a potential role in pathogenesis (21). Recently, there has been increased emphasis on correcting rotational deformities due to their association with recurrent deformities. Okuda et al. (6) studied the relationship between first metatarsal pronation, indicated by the shape of the first metatarsal head in plain films, and the recurrence of angular deformity (HVA ≥ 20 degrees) following operation (mean, 48 months; range, 14–125 months). A positive round shape, defined as abnormal pronation of the first metatarsal bone, in the early postoperative period correlated with a greater risk of increased angulation of the hallux in the late follow-up period [odds ratio (OR), 12.71; 95% CI, 3.21–50.36]. Ono et al. (23) evaluated the correlation between the shape of the first metatarsal head and the presence of sesamoid-metatarsal joint osteoarthritis in radiographs, finding a higher prevalence of osteoarthritis in round (77%) compared to intermediate (27%) and angular (29%) shapes (OR 22.9; *P*-value < .001). Another study by Shibuya et al. (5) used the tibial sesamoid position as a parameter representing degrees of first metatarsal rotation. This parameter was defined by Hardy and Clapham (8), and consists of 7 levels of grading, with levels higher than 4 indicated as abnormal. The tibial sesamoid position was found to be associated with early loss of hallux valgus correction, defined by an increased HVA of at least 3 degrees postoperatively (OR, 1.4; 95% CI, 1.10 to 1.85). While these studies indicated a connection between rotational parameters in radiographs and the recurrence of deformities or osteoarthritic changes in hallux valgus, the findings did not directly correlate with the functional or clinical outcomes of patients.

Conti et al. (7) studied 39 hallux valgus patients and found that those with decreased first metatarsal pronation postoperatively showed significant improvements in the physical function subscale of the Patient-Reported Outcomes Measurement Information System (PROMIS) (*P*-value = .007) and had lower rates of recurrent deformity (HVA ≥ 20 degrees) (*P*-value = .039) compared to patients with no change/increased pronation. However, no significant differences were observed in PROMIS pain interference (*P*-value = .380) and the pain intensity subscale (*P*-value = .443) between groups. Another study by An et al. (24) demonstrated postoperative improvements in PROMIS physical function, pain interference, pain intensity, and global physical health following correction with either plate and screw or cross-screw fixation. First metatarsal pronation, evaluated using the triplanar angle of pronation method (25), significantly improved with both fixation techniques. However, both preoperative and postoperative weight-bearing CT data were available for only about 70% of patients, limiting direct investigation of the association between rotational alignment and clinical outcomes. These findings align with those of our study, emphasising the importance of correcting metatarsal pronation in hallux valgus to achieve favourable clinical outcomes. In our study, the normal first metatarsal rotation following operation was associated with superior functional scores, improved other complaint subscales, and higher overall score on the VAS-FA, as well as better alignment subscales of the AOFAS Hallux MTP-IP.

There was a lack of direct evidence comparing the relationship between postoperative residual parameters and clinical outcomes in patients who underwent unilateral and bilateral hallux valgus operations. Most existing studies compare clinical outcomes or radiographic parameters between patients who had undergone unilateral and bilateral operations. For example, Gordon et al. (26) evaluated patients undergoing Minimally Invasive Chevron Akin osteotomy for hallux valgus correction, comparing unilateral and bilateral procedures. They reported significant improvements in the MOXFQ scores at two years postoperatively in both groups (*P*-value < .001), with no statistically significant differences in outcomes or postoperative IMA and HVA between groups. Similarly, Saragas et al. (27) conducted a retrospective study in patients who underwent hallux valgus osteotomy using the AOFAS scale and found significant improvements from the preoperative state to postoperative state in both unilateral and bilateral groups (*P*-value < .0001). There were no significant differences in postoperative outcomes (*P*-value = .95) or radiographic parameters (HVA: *P*-value = .32; IMA: *P*-value = .91) between groups.

Our study had several limitations. First of all, our study included only postoperative data due to the relatively recent adoption and limited availability of WBCT scans, particularly preoperatively at our institution. This restriction limited our ability to analyse the association between the degree of angular correction after surgery and clinical outcome improvement, hindering causal inferences. While WBCT offers detailed three-dimensional imaging, its routine use in hallux valgus evaluation remains debated due to the higher costs involved and the need to balance its benefits against conventional radiography. Second, we used a single parameter, the *α* angle, to evaluate first metatarsal rotation, as there is currently no standardised measure for first metatarsal pronation in WBCT. The *α* angle has shown efficacy in distinguishing the severity of first metatarsal rotation between hallux valgus patients (21.9 ± 6 degrees) and control patients (13.8 ± 4.1 degrees) (*P*-value < .001) (10). Third, the relatively small sample size may limit our ability to detect differences in certain clinical outcomes, potentially explaining inconsistent findings, such as those seen in the pain subscale. Small sample sizes reduce statistical power and increase the risk of type II error, wherein true differences may remain undetected. Moreover, due to the small sample size, formal tests for homogeneity of variance (e.g., Levene's test) were not performed, which could affect the robustness of parametric test assumptions. This limitation is acknowledged, and future studies with larger cohorts are needed to confirm our findings.

In addition, we did not apply formal adjustment for multiple comparisons, including comparisons across different operative procedures. Given that most of our results did not reach statistical significance, potentially influenced by the limited sample size, the adjustment methods were unlikely to alter results. Moreover, the uneven patient distribution across surgical procedures limited the robustness of analysis focused on this variable. Finally, the AOFAS Hallux MTP-IP includes physician-rated components assessed by a single surgeon, introducing potential risk of observer bias. While the AOFAS remains widely utilised, it is not entirely a patient-reported. We complemented it with validated self-reported outcome measures such as the FAOS and VAS-FA to provide a broader assessment of clinical outcomes. We acknowledge the limitations of the AOFAS and potential biases associated with subjective clinical assessments. In addition, our outcome evaluations were primarily based on clinical assessment, and objective biomechanical measurements such as gait analysis and foot pressure were not included, representing a limitation of this study.

The strengths of this study include a multidisciplinary team of assessors specialising in musculoskeletal radiology and orthopaedics, ensuring comprehensive expertise in data interpretation. We employed widely accepted and validated clinical assessment tools for hallux valgus (12–17), which improved the reliability and robustness of our findings. In addition, the use of multiple evaluation instruments allowed for a more thorough assessment of clinical outcomes. To our knowledge, this study is among the first to directly analyse the association between postoperative residual angular parameters and clinical outcomes, providing valuable insights that could enhance follow-up assessments in clinical practice.

## Conclusion

Our study highlights a notable association between residual pronation of the first metatarsal in the postoperative phase and poorer clinical outcomes, particularly in the function and other complaint subscales, the overall score of the VAS-FA, and the alignment subscale of the AOFAS Hallux MTP-IP. Despite these observed differences, no statistically significant disparities were found in the pain and function subscales of the AOFAS Hallux MTP-IP, the pain subscale of the VAS-FA, or any subscales of the FAOS. These mixed findings suggest a complex relationship between residual first metatarsal pronation and clinical outcomes. Consequently, while our results indicate a potential correlation between suboptimal postoperative function and satisfaction, further prospective studies with comprehensive preoperative and postoperative assessments are needed to elucidate causality and clarify these associations.

## Data Availability

The datasets presented in this article are not publicly available due to confidentiality concerns and institutional policies, but may be available from the corresponding author under specific conditions. Requests to access the datasets should be directed to the corresponding author.
